# Advanced Genomic Data Mining

**DOI:** 10.1371/journal.pcbi.1000121

**Published:** 2008-09-26

**Authors:** Xosé M. Fernández-Suárez, Ewan Birney

**Affiliations:** European Bioinformatics Institute, Ensembl Group, Wellcome Trust Genome Campus, Hinxton, Cambridge, United Kingdom; Whitehead Institute, United States of America

## Introduction

As data banks increase their size, one of the current challenges in bioinformatics is to be able to query them in a sensible way. Information is contained in different databases, with various data representations or formats, making it very difficult to use a single query tool to search more than a single data source.

**Figure 1:**
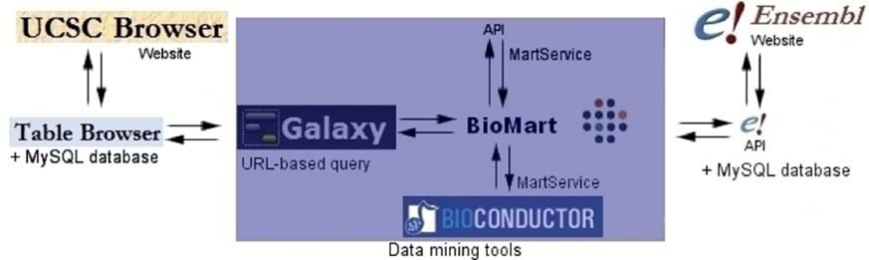
Diagram depicting the way different applications interact with data mining tools. The boxed area shows the topics discussed in this paper. BioMart's Central Server coordinates access to different databases: on the left-hand side, Galaxy interacts with BioMart by means of URL-based queries, allowing interaction with the UCSC Genome Browser and its Table Browser; while on the right interaction with Ensembl and its API is shown. Other platforms such as BioConductor rely on Web services (*MartService*) to retrieve information from the BioMart system. Additionally, BioMart is compliant with the DAS protocol [34] and can be queried by means of a Perl API, *biomart-perl*, as well as a Java API, *martj*.

Data mining is vital to bioinformatics as it allows users to go beyond simple browsing of genome browsers, such as *Ensembl* [1],[2] or the *UCSC Genome Browser* [3], to address questions; for example, the biological meaning of the results obtained with a microarray platform, or how to identify a short motif upstream of a gene, amongst others. There are a number of integrated approaches available, some of which are described below (Figure 1).

**Figure 2:**
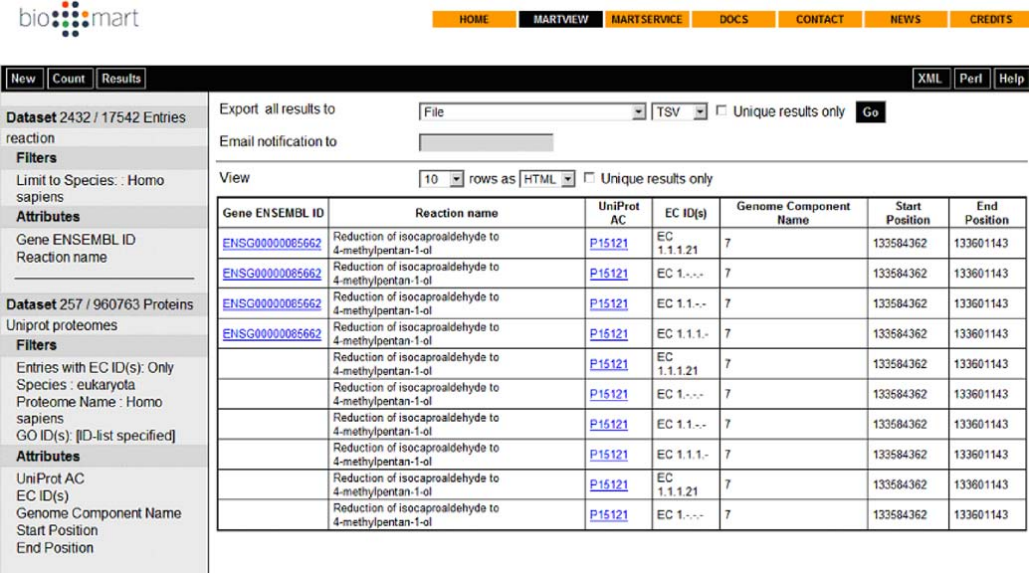
BioMart can join different datasets, in this case Reactome and UniProt to identify enzymes involved in carbohydrate metabolism.

The Table Browser at UCSC [4] supports text-based batch queries to the UCSC Genome Browser, limiting the output to entries meeting the selected criteria. A disadvantage of this tool is that users need to be familiar with the underlying database schema in order to know where their data is stored. Similarly, performing complex queries might require multiple steps that can be burdensome with this tool. Galaxy [5] provides a set of tools that can retrieve data from the Table Browser (Table Browser and BioMart will be explained below), facilitating complex queries that require multiple joins (Figure 2).

BioMart provides a query-oriented data management system to interact with different datasets (Ensembl [2], RGD [6],[7], and WormBase [8], among many others). This data “warehouse” was originally developed for Ensembl, creating EnsMart [9],[10]. From there, it was first deployed across the European Bioinformatics Institute (EBI), and now it has become a joint project between EBI and Cold Spring Harbor Laboratory (CSHL). The generic query system has shifted toward a federated approach that has been deployed for several biological databases, and has become a component of the Generic Model Organism Database (GMOD) project.

In this contribution, we provide some solutions for data mining; we focus on advanced ways of interacting with BioMart using other applications to retrieve information through different platforms such as Galaxy [5] and the *biomaRt* package of BioConductor [11],[12]. Many of these tools also interact with the UCSC Table Browser and have similar approaches using the UCSC system. We also address programmatic access using BioMart's own implementation of Web services (MartService). For local deployment of BioMart, see Table 1.

**Table 1:** URLs for additional information.

**BioMart documentation**	http://www.biomart.org/user-docs.pdf
**BioMart tutorials**	http://www.ensembl.org/info/using/website/tutorials/
	http://www.ensembl.org/common/Workshops_Online
**BioMart Central Server**	http://www.biomart.org/
**BioConductor**	http://www.bioconductor.org/
	http://www.bioconductor.org/packages/release/Software.html
**R**	http://www.r-project.org/
**Installing R**	http://cran.r-project.org/doc/manuals/R-admin.html
**R Archive Network**	http://cran.r-project.org/mirrors.html
**biomaRt documentation**	http://www.bioconductor.org/packages/1.8/bioc/vignettes/biomaRt/inst/doc/biomaRt.pdf
**Galaxy**	http://main.g2.bx.psu.edu/

## BioMart Web Interface

First we will focus on BioMart's Web interface (http://www.biomart.org) to illustrate how to join two different datasets: Reactome [13], a database of metabolic pathways, and UniProt [14], a catalogue of protein information. In this example, we need to obtain a catalogue of enzymes involved in carbohydrate metabolism in humans, as we are interested in a congenic disorder in this pathway. To ask this question without an integrated data mining tool, one would have to start with Reactome to find enzymes involved in reaction pathways in human and then compare those enzymes to a list of entries in UniProt. However, BioMart allows us to join the two databases.

We can start our query by clicking on ‘MartView’ from the Web interface at http://www.biomart.org, and selecting the Reactome database. Now, select the *reaction* dataset. Filters applied will be simply ‘Limit to Species’ *Homo sapiens*. Attributes can be selected as “*Reaction name*” and “Gene *ENSEMBL ID*”. At this stage, 2,432 entries meet our criteria (i.e. we have asked for all human reaction pathways in the Reactome database). Click on the ‘count’ button at the top to obtain this number.

Next, we can enrich our search for enzymes in the UniProt database. This will require the ‘linked’ or secondary dataset. Follow this description, or view the tutorials for use of the linked database at http://www.ensembl.org/common/Workshops_Onlineid117.

Click on the second ‘Dataset’ option at the left of the page. Select ‘UniProt proteomes’ as the database. In this instance, we will add as a filter the Gene Ontology (GO) [15] term ‘GO:0005975’ (associated with carbohydrate metabolic processes); this will be under ‘EXTERNAL IDENTIFIERS’, ‘Limit to proteins…GO ID(s)’ in the secondary dataset. Also select, under ‘External references’: ‘*Entries with EC ID(s)*’, to limit our query to enzymes only, and ‘*eukaryota*’ along with ‘*Homo sapiens*’ under ‘SPECIES’ (Species and Proteome Name, respectively). This will give a count of 257 in the secondary dataset. The genome location can be displayed in the output by choosing the following *Attributes*: “*Genome component name*” for the chromosome, “Start Position” and “End Position” for the coordinates. Click ‘Results’ for the table in Figure 2.

Now you have a list of enzymes in UniProt involved in carbohydrate metabolism in humans.

## BioConductor

BioConductor is open source software for the analysis of genomic data. It is based on the R language [16] (which is an implementation of the S language, a statistical programming language originally developed at Bell Laboratories to support research and data analysis of large statistical projects [17]).

R is an integrated software environment for data manipulation, which can be used as a statistics system (throughout many different packages). There are a large number of biologically relevant modules in BioConductor, some of which are described in [12] and at http://www.bioconductor.org/packages/release/Software.html. The *biomaRt* package provides an API (Application Programming Interface) in the scripting language R, allowing interaction with *biomaRt* databases. These include Ensembl, which produces and maintains automatic annotation on selected eukaryotic genomes; VEGA [18], the manually annotated Vertebrate Genome Annotation database; dbSNP [19], the Single Nucleotide Polymorphism database of NCBI; Gramene [20], a resource for comparative grass genomics; WormBase [9], the canonical database for *Caenorhabditis elegans* and related nematodes RGD [6],[7]; and Reactome [13], a curated knowledgebase of biological pathways, amongst others.

R can be installed on different platforms; there are binaries available for Unix, Windows, and Macintosh. For a list of the Comprehensive R Archive Network (CRAN), go to http://cran.r-project.org/mirrors.html. Once obtained, the source should be unpacked and installed following the instructions provided. There is a built-in help facility invoked with **help**, for instance ‘**help(debug)**’ will provide documentation about the **debug** function. Furthermore, following the installation of a package, a pre-built help search index is created. To know what commands are available in *biomaRt* use ‘**help.search(“biomaRt”)**’.

Once R is installed and compiled, the default set of BioConductor packages is easily installed using the **biocLite.R** installation script as follows:

**>source(“http://bioconductor.org/biocLite.R”)**

**>biocLite(“biomaRt”)**

With *biomaRt* installed, load the relevant library with the **library(biomaRt)** command, and then connect to any public BioMart database. The **listMarts** function will show which BioMart services are available. BioMart is structured in tables with *attributes* (the information you want to know) and *filters* (the information you know). You need to select a dataset (e.g., **rnorvegicus_gene_ensembl**), if you are interested in rat gene annotation from Ensembl. Issuing the following command: **rat = useMart(“ensembl”, dataset = “rnorvegicus_gene_ensembl”)**, would set the dataset queried to be the Ensembl rat genes.

**Figure 3:**
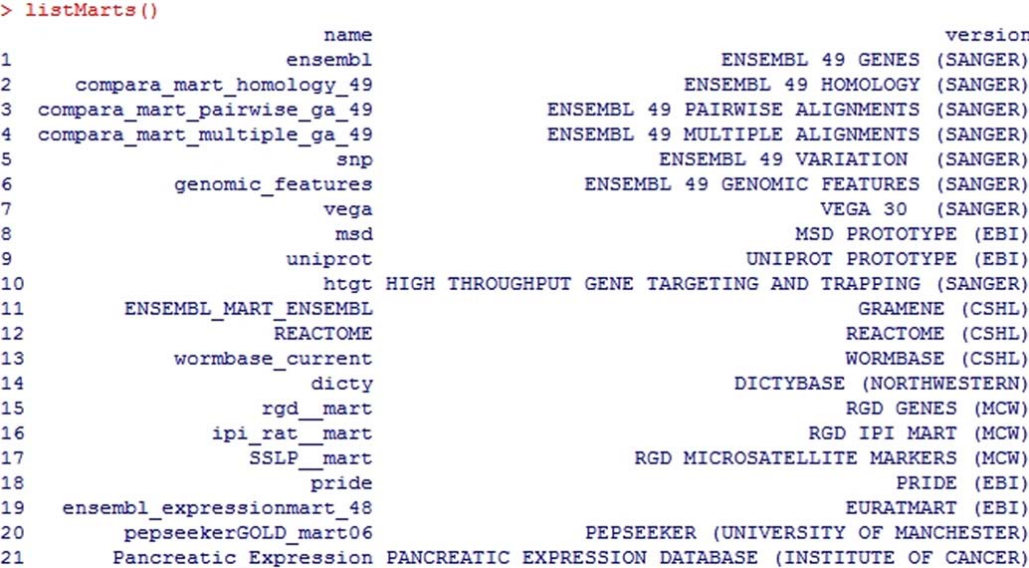
BioMart libraries available in the Central Server.

Below are two commands to query the library to see the currently available marts and datasets on the central server (Figure 3).

**>library(biomaRt)**

**>listMarts()**

Additionally, the **useMart** function allows you to select the relevant BioMart dataset, using the name provided by **listMarts**.

**>ensembl = useMart(“ensembl”)**

**>listDatasets(ensembl)**

**dataset version**

**1 oanatinus_gene_ensembl OANA5**

**2 gaculeatus_gene_ensembl BROADS1**

….…

Once you know which databases are available, select your dataset, which can be queried with **getBM(biomaRt)**. To finish the session, use **martDisconnect(biomaRt).**

Functions available allow extraction of identifiers from different sources including Ensembl IDs, several microarray platforms, UniProt, RefSeq [21], and EntrezGene [22]. Genome sequences can be retrieved by specific chromosomal coordinates for a given species, allowing a user to mine regions they define. For example, a user could view all annotations upstream of a differentially expressed gene in order to investigate putative regulatory elements. Similarly, *compara_mart_homology_47* supports queries across different species in order to identify homologous genes.

To illustrate how to use this tool, we provide an example: if you were interested in all mouse protein coding genes on Chromosome 10 along with their Ensembl and MGI identifiers, the following series of commands would carry out this query:

**>library(biomaRt)**

**>listMarts()**

**>ensembl = useMart(“ensembl”)**

**>listDatasets(ensembl)**

**>mouse = useMart(“ensembl”, dataset = “mmusculus_gene_ensembl”)**

**>getBM(c(“ensembl_gene_id”, “ensembl_transcript_id”, “mgi_symbol”), filters = “chromosome_name”,values = 10, mart = mouse)**

The output could be saved to a file *gene.ids*, which can be invoked by simply typing **“->gene.ids”**.

Researchers use DNA microarrays to establish the expression profiles of thousands of genes in a single experiment. Microarrays in their different incarnations have been used in a wide range of applications, e.g., disease characterization [23],[24] and identification of novel genes or gene regulatory networks [25].

A more specific use of *biomaRt* involving the recently developed CodeLink Rat Whole Genome Bioarrays (a platform from Applied Microarrays which features approximately 34,000 transcript and EST targets) will show how to further analyse data obtained from these bioarrays. The *Codelink* [26] R package can be installed (**biocLite(“codelink”)**). To obtain plotting functionality in the R statistical computing environment (e.g., **plotMA**, **plotCorrelations**, **plotDensities**, etc.), CodeLink bioarrays have been successfully applied to the identification of molecular signatures in colon cancer development [27]. In this paper, we will use some of the up-regulated probes from this study to illustrate how annotation could be retrieved using *biomaRt*, focusing on Ensembl genes associated to the probes. Data was obtained from the matrix plot of gene expression values (the authors provide supplementary data online at http://dnguyen.ucdavis.edu/.html/datatest3/main.html).

**>library(biomaRt)**

**Loading required package: XML**

**Loading required package: RCurl**

**>**

**>rat = useMart(“ensembl”, dataset = “rnorvegicus_gene_ensembl”)**

**Checking attributes and filters … ok**

Character strings are inserted using double (“) or single (‘) quotes, while the **c()**function is used to concatenate arrays.

**>upregulated = c(“AF003944”, “U67136”, “J04597”, “AF281635”, “BC090354”, “AF281635”, “BC090354”, “U12309”, “M14105”, “M14104”, “BC088159”, “D84450”, “BC061719”, “D90404”, “M26855”, “M26854”, “L00320”, “L00313”, “L00314”, “L00315”, “L00316”, “L00317”, “L00318”, “L00319”, “M11251”, “J00719”, “M37134”, “M26855”, “M26854”, “J00728”, “J00720”, “J00721”, “J00722”, “J00723”, “J00724”, “J00725”, “J00726”, “M34452”, “K00996”, “K01626”, “K01721”, “D00250”, “M13234”, “M13650”, “M26853”, “M19972”, “X63545”, “X12355”, “D63378”, “BC062393”)**

Given a list of expected ‘up-regulated’ mRNAs (EMBL or GenBank IDs), we can retrieve Ensembl Transcript IDs and the associated probes from CodeLink bioarrays, with their chromosome coordinates as shown in Table 2.

**Table 2:** R output (using *biomaRt* library) providing CodeLink bioarray IDs and their mappings to Ensembl transcripts (*chr: start-end position*), as explained in the text.

codelink	ensembl_transcript_id	embl	chromosome_name	start_position	end_position
GE13154	ENSRNOT00000014152	AF003944	1	125280974	125293051
GE13549	ENSRNOT00000018050	AF281635	4	152975181	152979366
GE20053	ENSRNOT00000015476	BC061719	8	101405877	101437760
GE20496	ENSRNOT00000020478	BC062393	3	108216369	108240138
GE19851	ENSRNOT00000015723	BC088159	16	17591820	17597859
GE13549	ENSRNOT00000018050	BC090354	4	152975181	152979366
GE1195465	ENSRNOT00000028196	D00250	1	81345425	81359415
GE20496	ENSRNOT00000020478	D63378	3	108216369	108240138
GE20053	ENSRNOT00000015476	D84450	8	101405877	101437760
GE20338	ENSRNOT00000022342	D90404	1	144629802	144661183
GE21631	ENSRNOT00000022342	D90404	1	144629802	144661183
	**ENSRNOT00000047540**	**J00719**	**1**	**81266845**	**81290470**
GE1195465	ENSRNOT00000028196	J00720	1	81345425	81359415
GE1195465	ENSRNOT00000028196	J00721	1	81345425	81359415
GE1195465	ENSRNOT00000028196	J00722	1	81345425	81359415
GE1195465	ENSRNOT00000028196	J00723	1	81345425	81359415
GE1195465	ENSRNOT00000028196	J00724	1	81345425	81359415
GE1195465	ENSRNOT00000028196	J00725	1	81345425	81359415
GE1195465	ENSRNOT00000028196	J00726	1	81345425	81359415
GE1195465	ENSRNOT00000028196	J00728	1	81345425	81359415
GE21002	ENSRNOT00000008416	J04597	6	98923111	98928505
GE1195465	ENSRNOT00000028196	K00996	1	81345425	81359415
GE1195465	ENSRNOT00000028196	K01626	1	81345425	81359415
GE1195465	ENSRNOT00000028196	K01721	1	81345425	81359415
	**ENSRNOT00000047540**	**L00313**	**1**	**81266845**	**81290470**
	**ENSRNOT00000047540**	**L00314**	**1**	**81266845**	**81290470**
	**ENSRNOT00000047540**	**L00315**	**1**	**81266845**	**81290470**
	**ENSRNOT00000047540**	**L00316**	**1**	**81266845**	**81290470**
	**ENSRNOT00000047540**	**L00317**	**1**	**81266845**	**81290470**
	**ENSRNOT00000047540**	**L00318**	**1**	**81266845**	**81290470**
	**ENSRNOT00000047540**	**L00319**	**1**	**81266845**	**81290470**
	**ENSRNOT00000047540**	**L00320**	**1**	**81266845**	**81290470**
	**ENSRNOT00000047540**	**M11251**	**1**	**81266845**	**81290470**
GE1195465	ENSRNOT00000028196	M13234	1	81345425	81359415
GE1195465	ENSRNOT00000028196	M13650	1	81345425	81359415
GE19851	ENSRNOT00000015723	M14104	16	17591820	17597859
GE19851	ENSRNOT00000015723	M14105	16	17591820	17597859
GE1195465	ENSRNOT00000028196	M19972	1	81345425	81359415
GE1195465	ENSRNOT00000028196	M26853	1	81345425	81359415
	**ENSRNOT00000034845**	**M26854**	**1**	**81266845**	**81290470**
	**ENSRNOT00000047540**	**M26854**	**1**	**81266845**	**81290470**
	**ENSRNOT00000034845**	**M26855**	**1**	**81266845**	**81290470**
	**ENSRNOT00000047540**	**M26855**	**1**	**81266845**	**81290470**
GE1195465	ENSRNOT00000028196	M34452	1	81345425	81359415
	**ENSRNOT00000047540**	**M37134**	**1**	**81266845**	**81290470**
GE20281	ENSRNOT00000014785	U12309	19	58601188	58628500
GE21915	ENSRNOT00000014785	U12309	19	58601188	58628500
GE21002	ENSRNOT00000008416	U67136	6	98923111	98928505
GE20496	ENSRNOT00000020478	X12355	3	108216369	108240138
GE20381	ENSRNOT00000041580	X63545	1	81780088	81853249
GE22156	ENSRNOT00000041580	X63545	1	81780088	81853249

**getBM(attributes = c(“codelink”, “ensembl_transcript_id”, “embl”, “chromosome_name”, “start_position”, “end_position”),filter = “embl”,values = upregulated, mart = rat)**

With this association between up-regulated probes and Ensembl annotation (Ensembl gene IDs could also be added), along with their location, additional annotation such as Gene Ontology terms could be included to obtain additional information about the putative function of the genes involved.

In this particular example, we can see that BioMart does not associate any probes to ENSRNOG00000033680 (ENSRNOT00000034845), number 40 in Table 2. Using the Ensembl Browser, we see this gene ID corresponds to Cyp2b15; if we have a closer look at this region (from Ensembl's ContigView or GeneView pages), we can see that this gene is very close to a gap in the current assembly (RGSC 3.4), and therefore Ensembl's independent mapping strategy failed to associate any CodeLink Bioarrays 30-mer oligonucleotide probes [28]. This demonstrates how the Ensembl graphical interface can enhance the understanding of the information obtained using BioMart.

## Other Data Mining Tools

**Figure 4:**
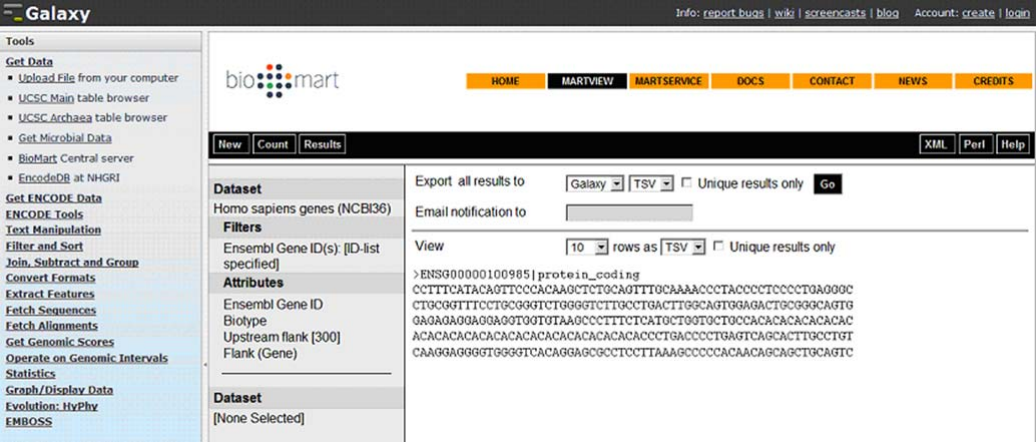
Screenshot displaying BioMart embedded in the Galaxy framework.

The number of applications available to perform analyses is rapidly growing, making it very difficult for users to know which tools would be suitable for them. Galaxy [5] provides a framework that integrates a variety of applications and data sources within an integrated workspace. Galaxy is available both through the Web (no installation required) and as a self-contained downloadable application that is easily customized [29]. Users can submit data to numerous computational tools (including 48 programs from the EMBOSS [30] suite, alongside tools for working with genomic sequences, alignments, and functional annotations). The results of analyses in Galaxy are stored remotely in ‘datasets’, which are accessible through the history panel on the right (collapsed in Figure 4). The history provides a complete record of an analysis, including all intermediate data, and can easily be shared with others. Galaxy also provides integration with the UCSC Table Browser tool in a similar way to its integration with the BioMart tool.

We can use Galaxy to search for a runt domain Runx2 motif (TGTGGTA) upstream of ENSG00000100985 (the human gene orthologous to ENSMUSG00000017737 where this transcription factor mediates induction of MMP9 [31]). Begin at the Galaxy interface (http://main.g2.bx.psu.edu). From the tools menu (left panel), select: **Get Data**>**BioMart** (Central Server) to query the ‘Ensembl genes’ database and retrieve 300 bp upstream (**Attributes**>**Sequences**>**Flank-coding region**) to ENSG00000100985. Following this, we can run **fuzznuc** (from EMBOSS) to find out if the Runx2 motif is upstream of the human gene. Such short motifs (length <10) cannot be identified by BLAST, SSAHA, or BLAT; that's why we will use EMBOSS.

**########################################**

**# Program: fuzznuc**

**# Commandline: fuzznuc**

**# -pattern TGTGGTT**

**# -pmismatch 1**

**# -complement no**

**########################################**

**# =  =  =  =  =  =  =  =  =  =  =  =  =  =  =  =  =  =  =  =  =  =  =  =  =  =  =  =  =  =  =  =  =  =  =  =  =  =  =**

**# Sequence: protein_coding from: 1 to: 300**

**# HitCount: 1**

**# Pattern_name Mismatch Pattern**

**# pattern1 1 TGTGGTT**

**#**

**# Complement: No**

**# =  =  =  =  =  =  =  =  =  =  =  =  =  =  =  =  =  =  =  =  =  =  =  =  =  =  =  =  =  =  =  =  =  =  =  =  =  =  =**

**Start End Pattern_name Mismatch Sequence**

**62 68 pattern1 1 TGCGGTT**

This example shows how Galaxy adds a layer of analysis to the genomic sequences retrieved from the UCSC Table Browser, or BioMart in this case (Figure 4). From the output of **fuzznuc**, we could expect a similar regulation for human MMP9, as we can find the Runx2 binding motif upstream of the human gene.

Galaxy offers users without large compute capacity the possibility of undertaking the analysis of multiple alignments (whole genome alignments are stored locally at the Galaxy site, compressed and indexed). One of Galaxy's strengths is the ease with which new tools can be integrated—new suites of tools for massively parallel sequence data, metagenomics, and statistical genetics are growing rapidly.

Web services provide an alternative way of integrating databases and tools (e.g., Taverna [32] and the *biomaRt* package of BioConductor), but users require some programming awareness. Galaxy removes this requirement; it relies on Web services to interact with external data sources such as BioMart and the UCSC Table Browser, providing a structured Web interface. Behind the scenes, when dealing with user data, jobs are wrapped and run in an abstract interface, to ensure reproducibility and avoid any problems associated with changes in the underlying Web services. For a more detailed description of Web services, see [33].

## Conclusions

We have seen how to go beyond simple browsing of data with data mining tools leveraging the BioMart system from different platforms, e.g., BioConductor (*biomaRt*), to find the association between microarray probe and Ensembl gene sets. Galaxy allowed us to use BioMart to extract information from Ensembl to identify some short motifs (beyond the threshold of BLAST detection) in the promoter region of a gene (MMP9). The Web interface of BioMart supports complex queries joining different datasets.

## Accession Numbers Used in the Text

GO IDs: GO:0005975

EMBL: AF003944, U67136, J04597, AF281635, BC090354, AF281635, BC090354, U12309, M14105, M14104, BC088159, D84450, BC061719, D90404, M26855, M26854, L00320, L00313, L00314, L00315, L00316, L00317, L00318, L00319, M11251, J00719, M37134, M26855, M26854, J00728, J00720, J00721, J00722, J00723, J00724, J00725, J00726, M34452, K00996, K01626, K01721, D00250, M13234, M13650, M26853, M19972, X63545, X12355, D63378, BC062393.

Ensembl Transcript IDs: ENSRNOT00000014152, ENSRNOT00000018050, ENSRNOT00000015476, ENSRNOT00000020478, ENSRNOT00000015723, ENSRNOT00000028196, ENSRNOT00000022342, ENSRNOT00000047540, ENSRNOT00000008416, ENSRNOT00000034845, ENSRNOT00000014785, ENSRNOT00000041580

Ensembl gene IDs: ENSRNOG00000033680, ENSMUSG00000017737, ENSG00000100985

CodeLink IDs: GE13154, GE13549, GE20053, GE20496, GE19851, GE1195465, GE20338, GE21631, GE21002, GE20281, GE21915, GE22156
